# User Performance Evaluation of Four Blood Glucose Monitoring Systems Applying ISO 15197:2013 Accuracy Criteria and Calculation of Insulin Dosing Errors

**DOI:** 10.1007/s13300-018-0392-6

**Published:** 2018-03-03

**Authors:** Guido Freckmann, Nina Jendrike, Annette Baumstark, Stefan Pleus, Christina Liebing, Cornelia Haug

**Affiliations:** Institut für Diabetes-Technologie, Forschungs- und Entwicklungsgesellschaft mbH an der Universität Ulm, Ulm, Germany

**Keywords:** Insulin dosing errors, ISO 15197, Lay-user, Self-monitoring of blood glucose, Surveillance error grid, System accuracy, User performance evaluation

## Abstract

**Introduction:**

The international standard ISO 15197:2013 requires a user performance evaluation to assess if intended users are able to obtain accurate blood glucose measurement results with a self-monitoring of blood glucose (SMBG) system. In this study, user performance was evaluated for four SMBG systems on the basis of ISO 15197:2013, and possibly related insulin dosing errors were calculated. Additionally, accuracy was assessed in the hands of study personnel.

**Methods:**

Accu-Chek^®^ Performa Connect (A), Contour^®^ plus ONE (B), FreeStyle Optium Neo (C), and OneTouch Select^®^ Plus (D) were evaluated with one test strip lot. After familiarization with the systems, subjects collected a capillary blood sample and performed an SMBG measurement. Study personnel observed the subjects’ measurement technique. Then, study personnel performed SMBG measurements and comparison measurements. Number and percentage of SMBG measurements within ± 15 mg/dl and ± 15% of the comparison measurements at glucose concentrations < 100 and ≥ 100 mg/dl, respectively, were calculated. In addition, insulin dosing errors were modelled.

**Results:**

In the hands of lay-users three systems fulfilled ISO 15197:2013 accuracy criteria with the investigated test strip lot showing 96% (A), 100% (B), and 98% (C) of results within the defined limits. All systems fulfilled minimum accuracy criteria in the hands of study personnel [99% (A), 100% (B), 99.5% (C), 96% (D)]. Measurements with all four systems were within zones of the consensus error grid and surveillance error grid associated with no or minimal risk. Regarding calculated insulin dosing errors, all 99% ranges were between dosing errors of − 2.7 and + 1.4 units for measurements in the hands of lay-users and between − 2.5 and + 1.4 units for study personnel. Frequent lay-user errors were not checking the test strips’ expiry date and applying blood incorrectly.

**Conclusions:**

Data obtained in this study show that not all available SMBG systems complied with ISO 15197:2013 accuracy criteria when measurements were performed by lay-users.

**Trial Registration:**

The study was registered at ClinicalTrials.gov (NCT02916576).

**Funding:**

Ascensia Diabetes Care Deutschland GmbH.

**Electronic supplementary material:**

The online version of this article (10.1007/s13300-018-0392-6) contains supplementary material, which is available to authorized users.

## Introduction

Accurate measurement results obtained with blood glucose (BG) monitoring systems intended for self-testing by diabetes patients are important for correct insulin dosing calculations and adequate therapeutic decisions [1–4]. Several self-monitoring of blood glucose (SMBG) measurements per day are recommended especially for patients on intensive insulin regimens [5]. Regular SMBG is also recommended for patients with less intensive insulin therapy [6].

Previous accuracy evaluations often analyzed accuracy of SMBG systems in the hands of professionals who are trained in the use of SMBG systems [7–10]. However, accuracy achieved by professionals does not necessarily reflect accuracy achieved by lay-users, i.e., people with diabetes [11–14]. The International Organization for Standardization’s (ISO) standard ISO 15197:2013, clause 8 [15], requires a user performance evaluation to assess if intended users are able to obtain accurate BG measurement results with an SMBG system. ISO 15197:2013 was harmonized with the regulations of the European Union as EN ISO 15197:2015 [16]. This harmonization had no impact on the requirements and procedures in ISO 15197:2013; changes were made to the foreword and an informative annex. According to ISO 15197 clause 8, a system’s user performance shall be evaluated with one test strip lot applying the following acceptance criteria: at least 95% of SMBG measurement results shall fall within ± 15 mg/dl (0.83 mmol/l) of comparison measurement results at glucose concentrations < 100 mg/dl (5.55 mmol/l) and within ± 15% at glucose concentrations ≥ 100 mg/dl (5.55 mmol/l).

In this study, accuracy of four SMBG systems in the hands of intended users was evaluated on the basis of testing procedures and acceptance criteria of ISO 15197:2013, clause 8. Accuracy was also evaluated in the hands of trained personnel. Potential insulin dosing errors were calculated by using a model described in detail by Baumstark et al. [17] in order to estimate possible influences of SMBG accuracy on glycemic control.

## Methods

The study was performed between September and October 2016 at the Institut für Diabetes-Technologie, Forschungs- und Entwicklungsgesellschaft mbH an der Universität Ulm (IDT), Germany. The study was conducted in compliance with the German Medical Devices Act, approved by the responsible Ethics Committee in Stuttgart, Germany and exempted from approval by the German Federal Institute for Drugs and Medical Devices. All procedures followed were in accordance with the ethical standards of the responsible committee on human experimentation (institutional and national) and with the 1964 Declaration of Helsinki, as revised in 2013. Informed consent was obtained from all patients for being included in the study.

## Subjects

To obtain at least 100 evaluable data sets, in total 158 subjects were enrolled. Table 1 shows demographic data for subjects included in data analysis. Included subjects did not use the investigated SMBG systems for the last 3 years according to their own statement.

**Table 1:** Subjects demographics

Included subjects	System A	System B	System C	System D
	*n* = 100	*n* = 100	*n* = 100	*n* = 100
Male, female (*n*)	54, 46	60, 40	59, 41	58, 42
Mean age (min–max) (years)	60.8 (29–80)	60.3 (27–79)	60.7 (25–80)	61.7 (25–80)
Diabetes type 1, 2 (*n*)	36, 64	35, 65	38, 62	36, 64
Education level (*n*):				
Secondary education grade	70	66	68	67
University entrance qualification	11	17	17	17
University degree	19	17	15	16
Time since diabetes diagnosis, mean (min–max) (years)	17.8 (1–66)	18 (1–55)	19.3 (1–66)	19.3 (1–66)
Subjects performing at least 1 SMBG measurement per week (*n*)	95	94	95	94

For each system, 100 different subjects were included in data analysis

Before study participation, a study physician reviewed the subjects’ self-reported medical history and medication, the inclusion and exclusion criteria for study participation (e.g., pregnancy or lactation period, severe acute disease, and/or chronic disease), and checked for interfering substances given in the manufacturers’ labelling.

## Test Systems

In this study, four SMBG systems were evaluated (Table 2): Accu-Chek^®^ Performa Connect (system A), Contour^®^ plus ONE (system B), FreeStyle Optium Neo (system C), and OneTouch Select^®^ Plus (system D). All systems were CE-marked. System B is a new system; systems A, C and D are available in Eastern Europe and Asia. All systems displayed plasma-equivalent glucose concentrations in milligrams per deciliter (mg/dl).

**Table 2:** System characteristics

System			Manufacturer’s comparison method	Enzyme test strips	Measurement conditions			Manufacturer
#	Name	Test strip lot (expiry date)			Temperature (°C)	Humidity (%)	Hematocrit (%)	
A	Accu-Chek Performa Connect	475098 (2017-09)	Hexokinase	Glucose dehydrogenase	8–44	10–90	10–65	Roche Diabetes Care GmbH, Mannheim, Germany
B	Contour plus ONE	DP6ELHD01C (2018-05)	Glucose oxidase	Glucose dehydrogenase	5–45	10–93	0–70	Bayer Consumer Care AG, Basel, Switzerland
C	FreeStyle Optium Neo	45001 67624 (2017-12)	Glucose oxidase	Glucose dehydrogenase	15–40	10–90	15–65	Abbott Diabetes Care Ltd., Witney, Oxon, UK
D	OneTouch Select Plus	3997403 (2017-08)	Glucose oxidase	Glucose oxidase	10–44	10–90	30–55	LifeScan Europe, Division of Cilag GmbH International Zug, Switzerland

Meters and test strips of system A were purchased from a pharmacy in Austria. For system B, five meters and test strips were purchased from a pharmacy in Poland; 115 meters were provided by the manufacturer. Meters of system C were bought online from amazon.co.uk, and test strips were purchased from a pharmacy in the UK. Meters and test strips of system D were purchased from a pharmacy in Germany.

Meters and test strips were stored, maintained, and used in compliance with the manufacturer’s labelling. Control measurements according to the manufacturer’s labelling were performed daily prior to the test procedure and for each test strip vial to ensure the proper function of each system.

Since, for systems B and D, the same meter was used for the familiarization and the measurement procedure (as described in Sect. “Test Procedure”), control measurements were also performed retrospectively after the measurement procedure to confirm the proper function of the system.

## Instructions for Use

According to ISO 15197:2013, clause 8, subjects shall be given the instructions for use and any training material that are routinely provided with the SMBG system.

For system A, C, and D, instructions for use routinely provided with the SMBG system were available. For system B, instructions for use were provided by the manufacturer. Since not all instructions for use were available in German, relevant information, i.e., preparation and performance of BG measurements, possible error sources, and error messages, was summarized in German in a comparable layout for each system.

## Comparison Measurement Methods

Comparison measurements were performed with a glucose oxidase (GOD) method (YSI 2300 STAT Plus glucose analyzer, YSI Incorporated, Yellow Springs, OH, USA) and a hexokinase (HK) method (Cobas Integra^®^ 400 plus, Roche Instrument Center, Rotkreuz, Switzerland). Comparison measurements were performed in duplicate with both comparison methods. Both methods provided BG values in milligrams per deciliter (mg/dl). For the YSI 2300 STAT Plus glucose analyzer and the Cobas Integra 400 plus instruments, conformity to traceability requirements of ISO 17511 [18] was confirmed by the manufacturers. Trueness and precision of the two analyzers were verified by regular internal and external quality control measures as required by the German national guideline (Rili-BÄK) [19]. In addition, daily quality control measurements were performed applying IDT internal standard operating procedures by using National Institute of Standards and Technology (NIST) standard reference material (SRM) 965b. The manufacturer’s comparison measurement method and/or the method used by the manufacturer for SMBG accuracy evaluation as indicated in the manufacturer’s labelling is shown in Table 2.

## Test Procedure

The test procedure was performed on the basis of ISO 15197:2013, clause 8, with one test strip lot for each system. For each subject, testing procedures were performed with three different SMBG systems; the evaluation of one system was completed before the evaluation of another system began. The number of SMBG systems used by the same subject was limited to three because the procedures were very time-consuming and attention-demanding for individual subjects. To minimize possible order effects, the systems were rotated according to a predefined pattern. The familiarization procedure (as described below) was performed with a designated training meter and the measurement procedure (as described below) with a designated test meter. Test meters were cleaned and disinfected after each subject. According to the systems’ labelling, system B and D might be infectious after use even if they were disinfected. For these systems, every subject got their own meter for the familiarization and the measurement procedure.

For each subject, the capillary blood’s hematocrit value was checked to be within the range indicated in the SMBG system’s labelling. For this purpose, samples were collected in heparinized capillaries and the capillaries were centrifuged. Hematocrit values were determined by using an alignment chart.

### Familiarization with SMBG Systems

As requested by ISO 15197:2013, each subject had the opportunity to review the instructions for use and to practice testing with a given system in a manner that represents how lay-users learn to use a new SMBG system. For this purpose, subjects were allowed to review the instructions for use and to perform up to three training measurements with control solution.

No additional instructions, training, and assistance were provided to the subjects. In addition, the familiarization procedure was supervised by study personnel to prevent any influence on a subject, e.g., by other subjects.

### Measurements

Measurements were performed in a controlled laboratory setting (temperature, 19.8–24.3 °C; humidity, 28.5–56.4%). Each subject was allowed to use the SMBG system’s instructions for use. Measurements were supervised by trained study personnel.

Each subject washed and dried their hands before the measurement procedure. Then, the subject collected his/her own capillary blood sample from a fingertip by skin puncture and performed a measurement using the test meter. For hygiene reasons, the skin puncture was performed with a single-use lancing device. For systems A, B, and D, study personnel removed each test strip from the vial and put it on the closed vial. For system C each test strip was packaged separately and was removed from the package by the subject. Test strip vials or packages were changed after approximately 10 subjects.

After the measurement with a given test system, subjects were asked if they thought that they performed the measurement correctly. Subjects repeated the measurement up to three times if they reported making a mistake or if no valid result was obtained. The first quantitative measurement result for which a subject did not report making a mistake in the measurement procedure was included in the accuracy analysis.

After the SMBG measurement by the subject, study personnel collected a capillary blood sample from the subject’s fingertip and performed measurements in duplicate with the same SMBG system, consecutively using the subject’s test meter and an additional meter. Before and after these duplicate SMBG measurements, samples for comparison measurements (first and second comparison measurement) were collected in lithium heparin tubes, aliquots were centrifuged within 7 min, and measurements were performed on separated plasma.

### Human Factors

The subjects’ measurement technique was observed and documented by study personnel with regard to the manufacturer’s labelling, i.e., preparation of the measurement, insertion of test strips, blood sampling, application of blood, and mistakes in device handling. Errors documented only by the study personnel but not by the subjects did not lead to exclusion from accuracy analysis.

## Data Analysis

### Data Exclusions

Data were excluded from analysis for the following reasons: the subject reported making a mistake in his/her measurement procedure and the measurement was repeated; the number of 100 valid measurement results was already reached; the subject obtained no valid result and the measurement was repeated; hemolysis in plasma samples for comparison measurements, errors regarding comparison measurements; quality control measurement results obtained with the comparison method were outside predefined limits or no valid measurement result was obtained; the difference between the first and second comparison measurements exceeded the acceptance criteria for sample stability (≤ 4 mg/dl at glucose concentrations < 100 mg/dl or ≤ 4% at glucose concentrations ≥ 100 mg/dl).

### User Performance Evaluation

For each system, data from 100 subjects (*n* = 100) were included in the evaluation. Accuracy was evaluated by comparing the SMBG system’s measurement result with the respective comparison measurement result (first comparison measurement) according to ISO 15197:2013, clause 8, by calculating the number and percentage of results within ± 15 mg/dl of the mean comparison measurement result at BG concentrations < 100 mg/dl and within ± 15% at BG concentrations ≥ 100 mg/dl. In addition, the percentage of results within the clinically acceptable zones A and B of the consensus error grid (CEG) and the bias (systematic measurement difference) according to Bland and Altman [20] were calculated.

Data are also presented in radar plots [21, 22], an alternative approach to visualize the measurement accuracy of an SMBG system, and in surveillance error grids (SEG) [23], which is an alternative approach to assess the clinical risk associated with SMBG systems’ accuracy.

### Evaluation of Human Factors

For each system, the occurrence of user errors in performing the measurement procedure was analyzed.

### Evaluation of Accuracy in the Hands of Study Personnel

For each system, data from 100 subjects (*n* = 200, duplicate measurements) were included in the evaluation. Accuracy was evaluated with one test strip lot for each system by comparing the study personnel’s SMBG measurement result with the respective mean comparison measurement result (first and second comparison measurement). The same parameters as for lay-users were calculated and are presented as described above.

### Calculation of Possibly Related Insulin Dosing Errors

All data included in the accuracy evaluation (lay-users *n* = 100, study personnel *n* = 200) were assessed regarding possibly related insulin doses based on the model described in detail by Baumstark et al. [17]. In this model, only short-term insulin doses (i.e., meal-related doses and doses for BG correction), but not long-term (basal) insulin doses were covered.

The model utilizes the mealtime insulin dosing formula used by the Diabetes Teaching Center at the University of California San Francisco (UCSF), San Francisco, CA [24]. Insulin doses were calculated for comparison method results and SMBG system results and the following fixed parameters: 60 g carbohydrate intake, 1 unit per 15 g insulin-to-carb ratio, a target BG value of 100 mg/dL, and an insulin sensitivity of 25 mg/dL per unit.

## Results

### Accuracy in the Hands of Lay-users

**Fig. 1:**
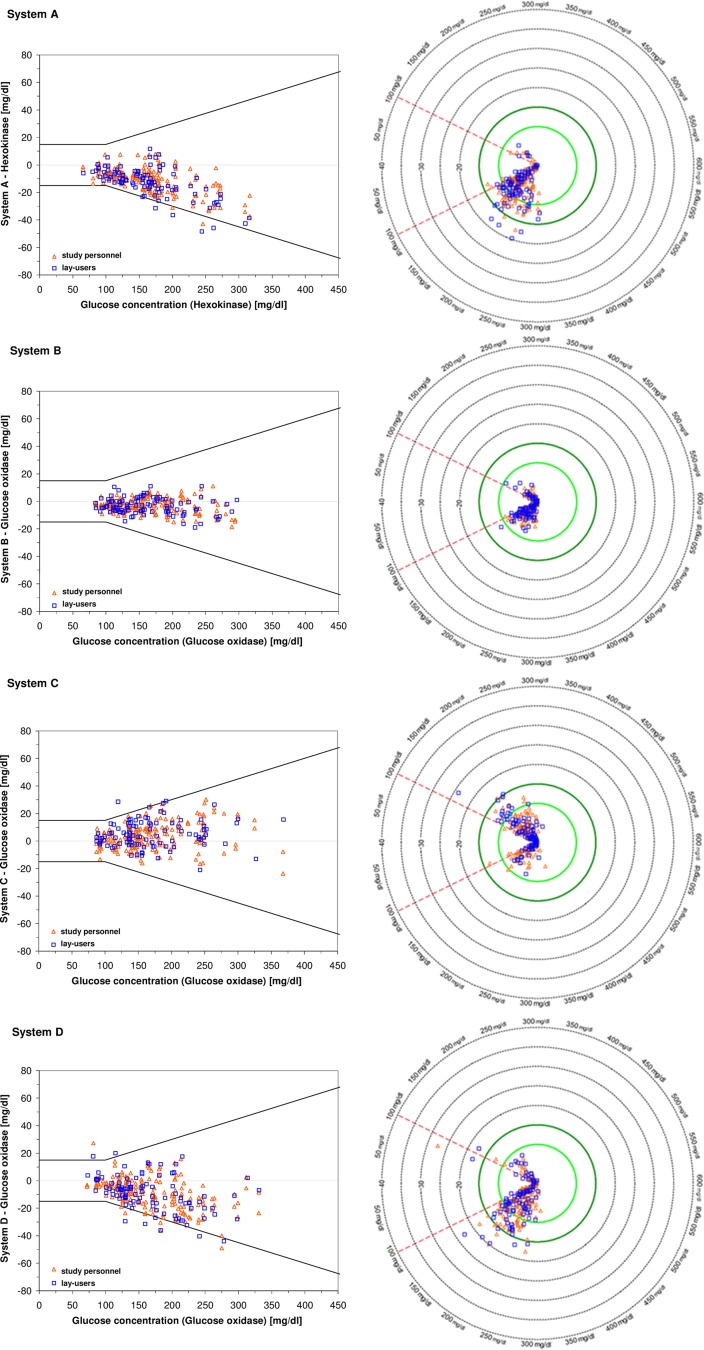
Difference plots (left side) and radar plots (right side) for the tested lot of each of the four SMBG systems when evaluated against the respective manufacturer’s comparison method (hexokinase for system A, glucose oxidase for system B, C, D). Measurements performed by lay-users (*n* = 100) are displayed in blue squares, measurements performed by study personnel (*n* = 200) are displayed in orange triangles. Difference plots: ISO 15197:2013 accuracy limits (± 15 mg/dl for BG concentrations < 100 mg/dl and ± 15% for BG concentrations ≥ 100 mg/dl) are displayed in solid lines. Radar plots: Data points show differences between SMBG measurement results and the respective comparison measurement result, absolute differences for BG concentrations < 100 mg/dl and relative differences for BG concentrations ≥ 100 mg/dl. The absolute values of the differences define the location of the data points, i.e., the distance from the center of the plot, and the sign of the differences indicates the hemisphere (positive sign, upper hemisphere; negative sign, lower hemisphere). The direction with respect to the center of the plot in which the data point lies depends on the comparison method result. In radar plots, high accuracy is represented by tightly grouped data points close to the center of the plot. The circle in dark green highlights the system accuracy limits of ISO 15197:2013 (± 15 mg/dl/± 15%)

**Fig. 2:**
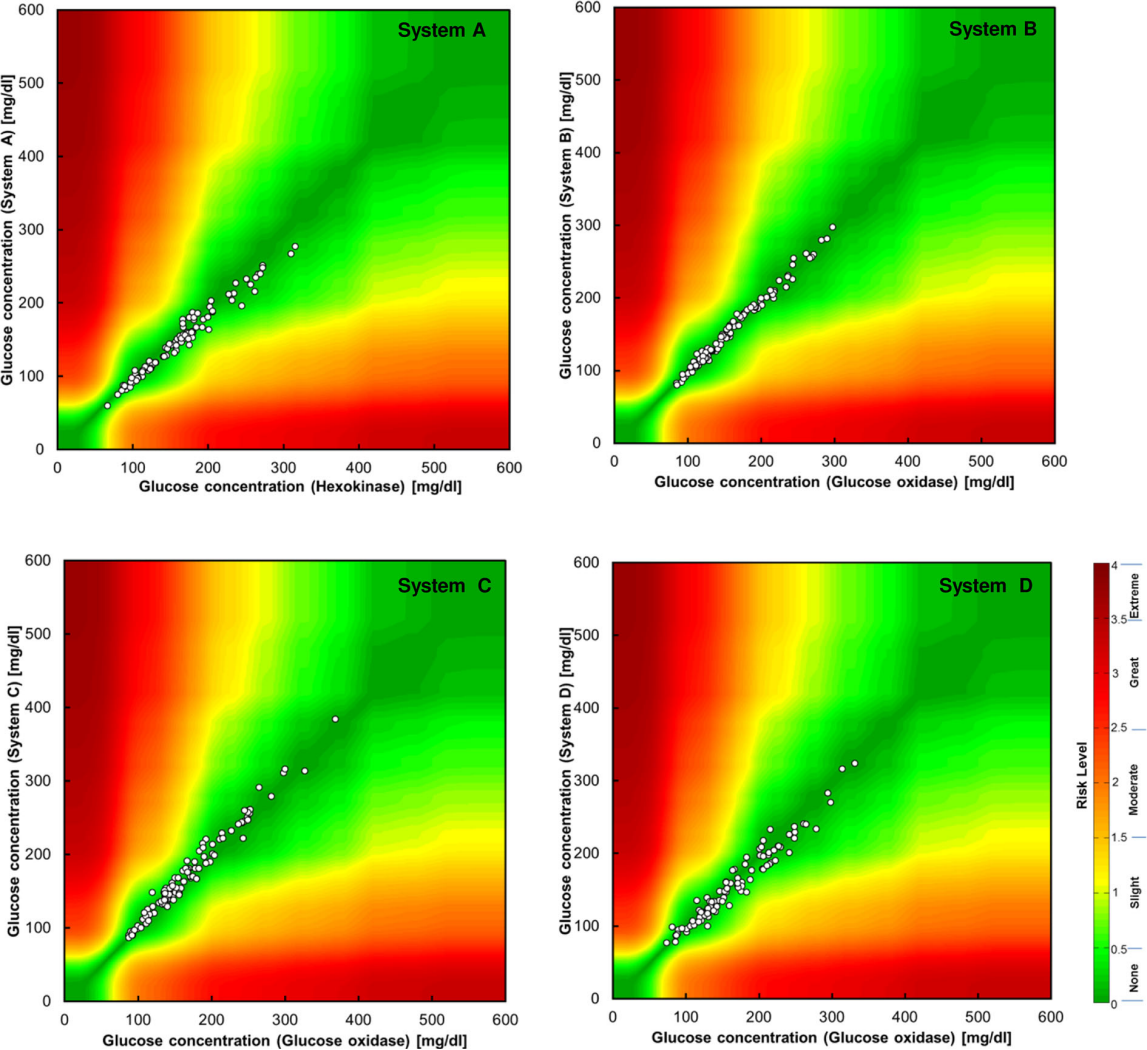
Surveillance error grid analysis for the tested test strip lot of each of the four SMBG systems. Data are shown for lay-user SMBG measurements (*n* = 100) evaluated against the respective manufacturer’s comparison method. Colors indicate associated risk levels ranging from none (dark green) to extreme (dark red)

Accuracy results for each system when evaluated against the manufacturer’s comparison method are presented in Table 3, Fig. 1 (difference plot and radar plot), and Fig. 2 (SEG). Results when evaluated against the alternative comparison method are provided in Supplemental Table 1 and Supplemental Fig. 1).

**Table 3:** Accuracy results based on ISO 15197:2013 criteria, results within consensus error grid (CEG) zones A and B, results within surveillance error grid zones with “no risk” or “slight lower risk”, relative bias according to Bland and Altman, and calculated insulin dosing error

System	User	Results within				Relative bias	Calculated insulin dosing error	
		±15 mg/dl/± 15%	CEG zones A + B	SEG zones				
				No risk	Slight, lower risk		Median (50th percentile)	99% ranges between the 0.5th and 99.5th percentile
				Risk score				
				0–0.5	> 0.5–1.0			
		%	%	n		%	Units	
A	Lay-users	96	100	100	0	− 7.6	− 0.9	− 2.7 to − 0.1
	Study personnel	99	100	197	3	− 6.8	− 0.8	− 2.4 to 0.0
B	Lay-users	100	100	100	0	− 2.2	− 0.3	− 1.0 to + 0.3
	Study personnel	100	100	200	0	− 2.2	− 0.2	− 1.0 to + 0.2
C	Lay-users	98	100	97	3	+ 2.8	+ 0.3	− 0.6 to + 1.4
	Study personnel	99.5	100	198	2	+ 1.6	+ 0.2	− 0.7 to +1.4
D	Lay-users	92	100	97	3	− 5.6	− 0.6	− 2.4 to +0.6
	Study personnel	96	100	193	7	− 5.3	− 0.7	− 2.5 to + 0.3

Results are shown for measurements performed by lay-users (*n* = 100) and by study personnel (*N* = 200) when evaluated by using the manufacturer’s comparison method (hexokinase for system A, glucose oxidase for systems B, C, and D)

**Fig. 3:**
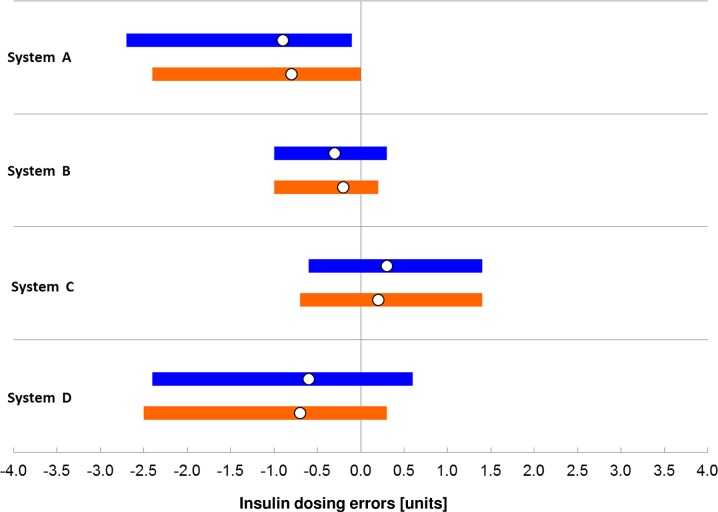
Modelled insulin dosing errors. Bars in blue (lay-users) and orange (study personnel) indicate ranges in which 99% of all dose errors were found, with the white circle showing the median dose error. Data are shown when evaluated against the respective manufacturer’s comparison method (hexokinase for system A, glucose oxidase for system B, C, D)

Three of the four systems fulfilled ISO 15197:2013 accuracy criteria in the hands of lay-users with the investigated test strip lot showing 96% (A), 100% (B), and 98% (C) of results within ± 15 mg/dl and ± 15% of the comparison method results at BG concentrations < 100 mg/dl and ≥ 100 mg/dl, respectively. One system (D) did not fulfill ISO 15197:2013 accuracy criteria with the investigated test strip lot showing 92% of results within the limits (± 15 mg/dl and ± 15%). All four systems showed 100% of results within CEG zones A and B. Regarding the SEG, all measurements with systems A and B were within the “no risk” zone (risk score 0–0.5). The relative bias ranged from − 7.6% (system A) to + 2.8% (system C) with a smallest bias of − 2.2% (system B). Median modelled insulin dosing errors ranged from − 0.9 units (system A) to + 0.3 units (system C) with all 99% ranges between dosing errors of − 2.7 and + 1.4 units (Table 3, Fig. 3).

### Human Factors

Frequently observed lay-user errors were not checking the test strip’s expiry date and incorrect blood application, e.g., application of an insufficient blood amount, pressing the fingertip onto the test strip, or applying the blood incorrectly onto the test field (Table 4). Because all test strips were used in this study before the expiry date, this error had no impact on measurement results.

**Table 4:** Human factors: number of errors in the measurement technique of 100 lay-users observed by study personnel

	Number of lay-user errors			
	System A	System B	System C	System D
Preparation of the measurement—test^a^ strip’s expiry date was not checked	90	97	87	101
Insertion of test strips—e.g., not inserted correctly, kinked, bent	7	4	5	4
Blood sampling—e.g., blood drop too small, too old, smeared	3	3	11	8
Blood application—blood was applied incorrectly, e.g., insufficient amount, fingertip was pressed onto the test strip; blood was not applied onto the test field correctly	17	20	38	35
Device handling—e.g., blood application before meter was ready for use, test strip was removed before completion of the measurement, fingertip was not removed from the test trip after the signal tone	8	6	12	7
Number of incorrectly performed measurements as reported by subjects	4	2	17	7

^a^Only non-expired test strips were used in this study; therefore, not checking the test strip’s expiry date had no impact on measurement results

### Accuracy in the Hands of Study Personnel

Accuracy results for one lot of each system when evaluated against the manufacturer’s comparison method are presented in Table 3 and Fig. 1 (difference plot and radar plot). Results when evaluated against the alternative comparison method are provided in Supplemental Table 1 and Supplemental Fig. 1.

All four systems fulfilled ISO 15197:2013 accuracy criteria in the hands of study personnel with the investigated test strip lot showing 99% (A), 100% (B), 99.5% (C), and 96% (D) of results within ± 15 mg/dl and ± 15% of the comparison method results at BG concentrations < 100 mg/dl and ≥ 100 mg/dl, respectively. All four systems showed 100% of results within CEG zones A and B. Regarding the SEG, all measurements with system B were within the “no risk” zone (risk score 0–0.5). The relative bias ranged from − 6.8% (system A) to + 1.6% (system C) with a smallest bias of + 1.6% (system C). Median modelled insulin dosing errors ranged from − 0.8 units (system A) to + 0.2 units (system C) all with 99% ranges between dosing errors of − 2.5 and + 1.4 units.

## Discussion

In this study, system accuracy of four SMBG systems in the hands of lay-users, i.e., diabetes patients, was evaluated with one test strip lot on the basis of testing procedures and accuracy criteria of ISO 15197:2013, clause 8. Additionally, accuracy in the hands of study personnel trained in operating the SMBG systems was evaluated with one test strip lot for each of the four systems. According to ISO 15197, user performance evaluation shall demonstrate that intended users are able to obtain accurate measurement results when operating the SMBG system, given only the instructions for use and training materials routinely provided with the systems.

Three out of four systems fulfilled accuracy criteria in the hands of lay-users with the evaluated test strip lot showing 96% (system A), 100% (system B), and 98% (system C) of results within ± 15 mg/dl for BG concentrations < 100 mg/dl and ± 15% for BG concentrations ≥ 100 mg/dl. All systems fulfilled ISO 15197:2013 accuracy criteria in the hands of trained study personnel. One system (B) showed 100% of results in the hand of lay-users and study personnel, and similar accuracy results for this system were obtained in a previous user performance evaluation [25].

The other three systems showed slightly better accuracy results for study personnel measurements compared to lay-user measurements. Different studies showed that accuracy achieved by trained study personnel is often better than accuracy achieved by lay-persons [11, 12, 26, 27]; however, only few user performance evaluations were performed on the basis of procedures described in ISO 15197:2013 [25, 26, 28–30].

For all four systems, all measurements were within zones of the CEG and SEG that are associated with no or minimal risk, irrespective of the user group. ISO 15197:2013, clause 6.3, requires a CEG analysis for system accuracy evaluation. The CEG is developed for type 1 diabetes patients and is based on data collected in 1994 [31]. The CEG is divided into eight zones indicating the estimated risk to the patient. The SEG also assesses the clinical risk but is based on much more recent survey data [23]. The SEG is designed as an assessment tool of SMBG systems’ postmarket risk. SEG risk zones are color-coded and data points are assigned to continuous individual risk scores.

For both comparison measurement methods used in our study, compliance with traceability requirements was confirmed by the manufacturers. In this study, the comparison measurement method/system applied had no impact as to whether the data obtained comply with ISO 15197:2013 accuracy criteria or not. However, differences in accuracy data depending on the applied comparison method/system were found which is consistent with results of two previous ISO 15197-based studies performed at our institute [9, 32]. This influence might be reduced if manufacturers would adjust calibration of their reference methods [33].

This study design has several limitations. According to ISO 15197:2013, the user performance evaluation shall also include the evaluation of the instructions for use and the messages displayed on the meter by a questionnaire in order to assess whether the instructions and messages are adequate and easy to understand. Since not all instructions for use were available in German, subjects received a summarized instruction for use (in German) for each system with relevant information for the preparation and performance of BG measurements. Therefore, instructions for use and the messages displayed on the respective meters were not evaluated. In this study, only one lot was evaluated on the basis of ISO 15197:2013, clause 8. According to ISO 15197:2013, clause 6.3, system accuracy evaluation in the hands of study personnel shall be performed with three test strip lots, since lot-to-lot variations between multiple test strip lots used for the same system can markedly affect a system’s accuracy results [11, 34, 35].

Regarding the subjects’ measurement technique, not checking the expiry date of test strips before the measurement was the most frequent error. It might be that subjects assumed that they were only provided with non-expired test strips. Because test strips were used in this study before their expiry date, this error had no impact on measurement results. However, different studies showed that the use of deteriorated test strips can affect SMBG measurement results [36, 37].

For all four systems, an incorrect blood application, e.g., applying an insufficient amount of blood, pressing the fingertip onto the test strip, or applying the blood not onto the test field, was the most frequent error during the measurement procedure. Since incorrect blood application can potentially affect SMBG measurement results, adequate SMBG training and education should be an integral part of diabetes therapy [36, 38].

Accurate SMBG measurement results are crucial for adequate insulin dosing decisions by patients on insulin therapy. Modelling analyses showed that inaccurate BG measurements can lead to insulin dosing errors [1, 3, 4, 39, 40] which can adversely affect glycemic control and increase the risk of long-term complications in patients on intensive insulin therapy. In this study, negative median insulin dosing errors (too small insulin doses) were calculated for three systems (A, B, D) and positive median insulin dosing errors (too high insulin doses) were calculated for one system (C). Except for one system (D), median calculated insulin dosing errors were larger when SMBG measurements were performed by lay-persons. In addition, the four systems showed differences in the width of the error range (99% ranges between the 0.5th percentile and the 99.5th percentile).

## Conclusions

In this user performance evaluation, three out of four systems fulfilled ISO 15197:2013 accuracy criteria with the investigated test strip lot. However, the systems showed slight differences in the number of results within ISO 15197:2013 accuracy limits. All four systems fulfilled ISO 15197:2013 accuracy criteria with the investigated test strip lot in the hands of study personnel, with three systems showing better accuracy results when measurements were performed by trained study personnel. One system (B) showed 100% of results within the ISO 15197:2013 accuracy limits in the hands of lay-users and in the hands of study personnel. In both user groups, calculated median insulin dosing errors were within ± 0.9 units and tended to be larger when measurements were performed by lay-users. Inaccurate SMBG measurements can result in insulin dosing errors and adversely affect glycemic control. Therefore, manufacturers should provide high quality SMBG systems that are easy to use and resistant to user errors.

## Electronic supplementary material

Below is the link to the electronic supplementary material.

Supplementary material 1 (PDF 847 kb)
