# Supplementary material for: User Performance Evaluation of Four Blood Glucose Monitoring Systems Applying ISO 15197:2013 Accuracy Criteria and Calculation of Insulin Dosing Errors
Source: Diabetes Ther. 2018 Mar 3;9(2):683–97. doi: 10.1007/s13300-018-0392-6 (PMC6104257; doi:10.1007/s13300-018-0392-6)
Supplement: Supplementary file 1 — Supplementary material 1 (PDF 847 kb) [file 13300_2018_392_MOESM1_ESM.pdf]

System A

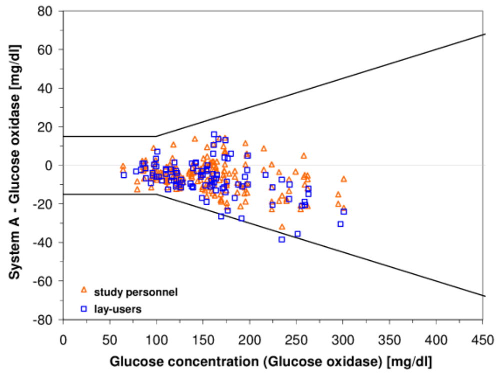


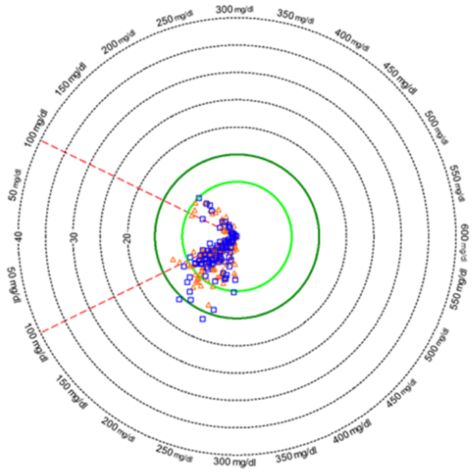

System B

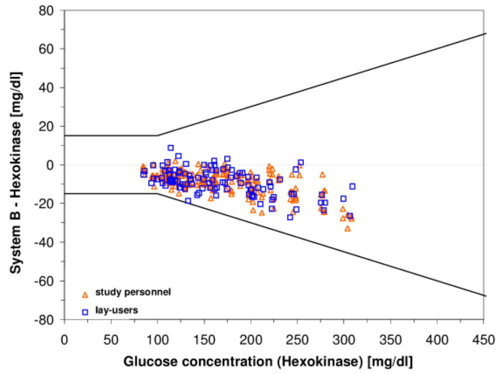


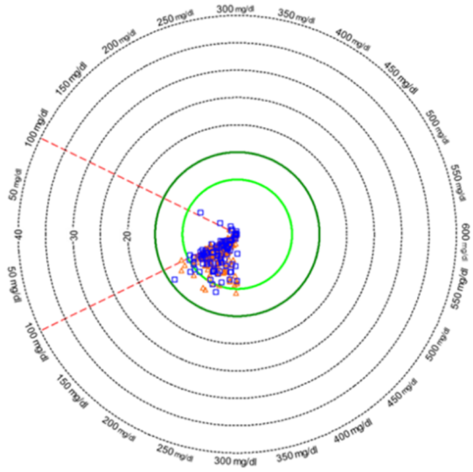

System C

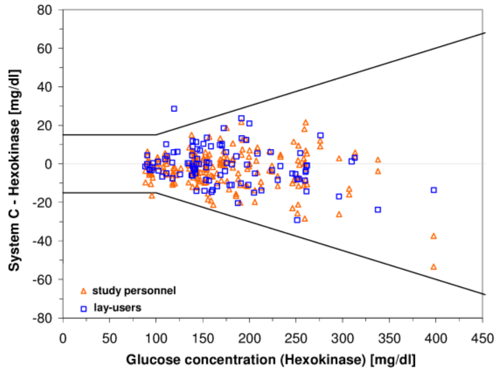


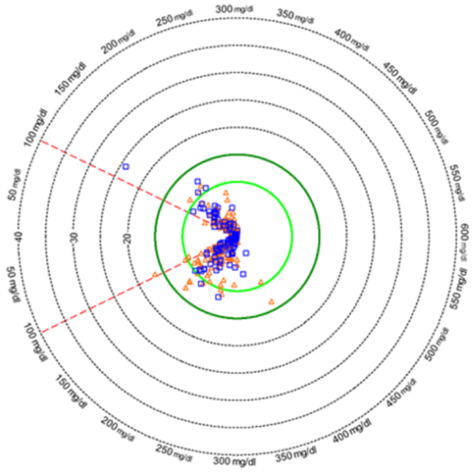

System D

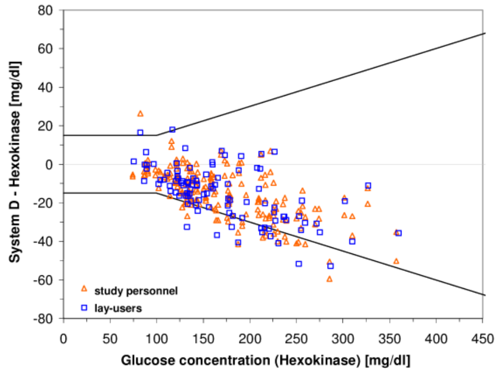


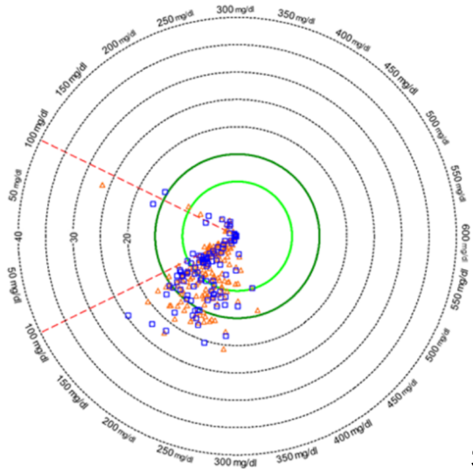

Supplemental

Figure 1. Difference plots (left side) and radar plots (right side) for the tested lot of each of the four SMBG systems when evaluated against the respective alternative comparison method (glucose oxidase for system A, hexokinase for system B, C, D). Measurements performed by lay-users (n=100) are displayed in blue squares, measurements performed by study personnel (n=200) are displayed in orange triangles. Difference plots: ISO 15197:2013 accuracy limits (±15 mg/dl for BG concentrations <100 mg/dl and ±15% for BG concentrations ≤100 mg/dl) are displayed in solid lines. Radar plots: Data points show differences between SMBG measurement results and the respective comparison measurement result, absolute differences for BG concentrations <100 mg/dl and relative differences for BG concentrations ≥100 mg/dl. The absolute values of the differences define the location of the data points, that is, the distance from the center of the plot, and the sign of the differences indicates the hemisphere (positive sign: upper hemisphere, negative sign: lower hemisphere). The direction with respect to the center of the plot in which the data point lies depends on the comparison method result. In radar plots, high accuracy is represented by tightly grouped data points close to the center of the plot. The circle in dark green highlights the system accuracy limits of ISO 15197:2013 (±15 mg/dl / ±15%). The direction with respect to the center of the plot in which the data point lies depends on the comparison measurement result.

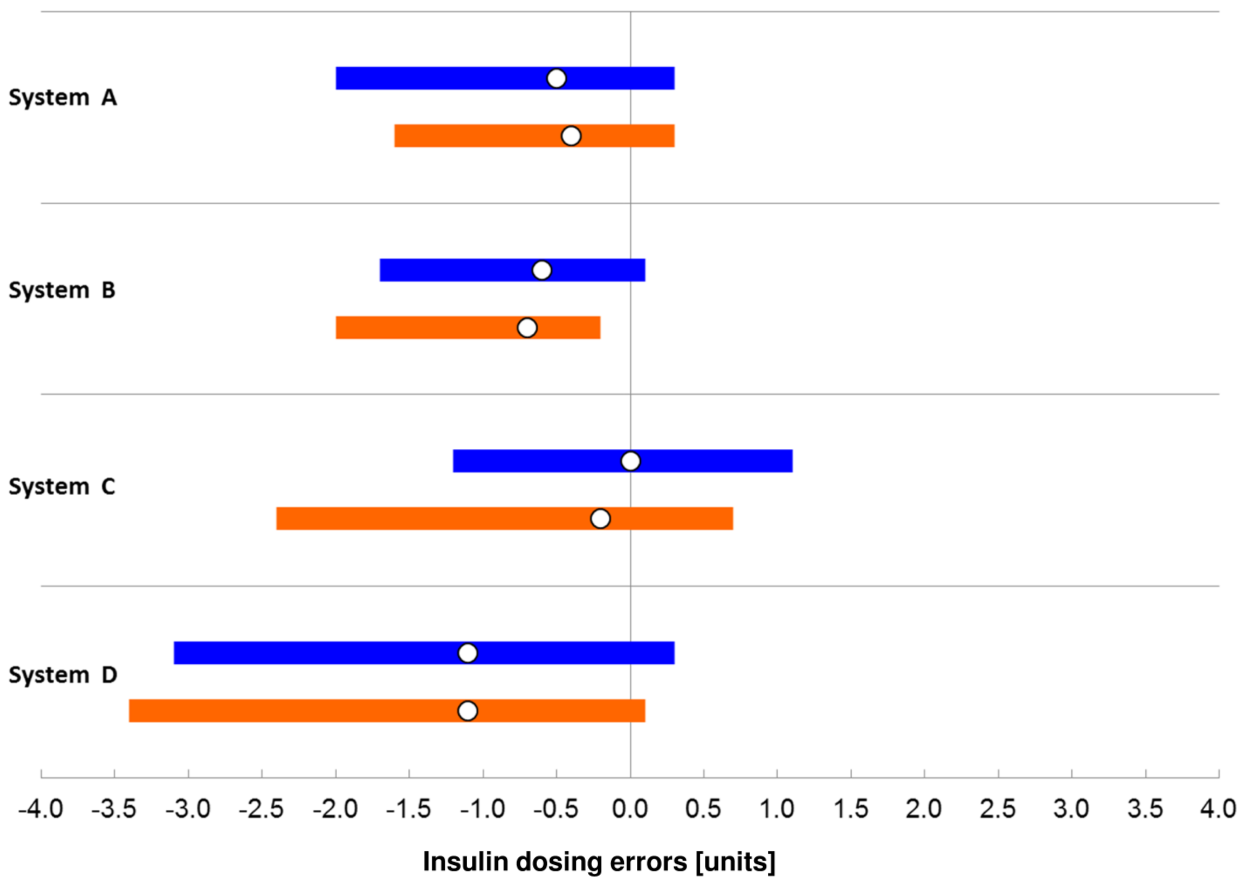


Supplemental Figure 2. Modelled insulin dosing errors. Bars in blue (lay-users) and orange (study personnel) indicate ranges in which 99% of all dose errors were found, with the white circle showing the median dose error. Data are shown when evaluated against the respective alternative comparison method (glucose oxidase for system A, hexokinase for system B, C, D).

Supplemental Table 1: Accuracy results based on ISO 15197:2013 criteria, results within Consensus Error Grid (CEG) zones A and B, results within Surveillance Error Grid zones with ‘no risk’ or ‘slight, lower risk’, relative bias according to Bland and Altman and calculated insulin dosing error. Results are shown for measurements performed by lay-users (n=100) and by study personnel (n=200) when evaluated by using the alternative comparison method (glucose oxidase for system A, hexokinase for systems B, C and D).

| System | User | Results within | | | | Relative bias | Calculated insulin dosing error | |
|---|---|---|---|---|---|---|---|---|
| | | ±15 mg/dl / ±15% | CEG zones A+B | SEG zones | | | Median (50th percentile) | 99% ranges between the 0.5th and 99.5th percentile |
| | | | | No risk | Slight, lower risk | | | |
| | | | | Risk score | | | | |
| | | | | 0-0.5 | >0.5-1.0 | | | |
| | | % | | | | % | Units | |
| A | Lay-users | 98 | 100 | 100 | 0 | -4.6 | -0.5 | -2.0 to +0.3 |
| | Study personnel | 100 | 100 | 199 | 1 | -3.7 | -0.4 | -1.6 to +0.3 |
| B | Lay-users | 100 | 100 | 100 | 0 | -5.4 | -0.6 | -1.7 to +0.1 |
| | Study personnel | 100 | 100 | 199 | 1 | -5.5 | -0.7 | -2.0 to -0.2 |
| C | Lay-users | 99 | 100 | 99 | 1 | -0.5 | 0.0 | -1.2 to +1.1 |
| | Study personnel | 99.5 | 100 | 196 | 4 | -1.9 | -0.2 | -2.4 to +0.7 |
| D | Lay-users | 84 | 100 | 94 | 6 | -8.8 | -1.1 | -3.1 to +0.3 |
| | Study personnel | 86 | 100 | 188 | 12 | -8.6 | -1.1 | -3.4 to +0.1 |
